# Supplementary material for: Relationships between Child Development at School Entry and Adolescent Health—A Participatory Systematic Review
Source: Int J Environ Res Public Health. 2021 Nov 4;18(21):11613. doi: 10.3390/ijerph182111613 (PMC8582847; doi:10.3390/ijerph182111613)
Supplement: Supplementary file 1 [file ijerph-18-11613-s001.zip › Additional file 3 Search Strategy Nov 2020.pdf]

Additional file 3: Search Strategy for Relationships between child development at school entry and adolescent health – a participatory systematic review

Exposure	Outcome	Relationship	Studies
"school readiness"	((health outcome* or health behavio* or "executive function" or anx* or depress* or "social competence" or "self-esteem" or wellbeing or obesity or weight or "mental health" or health* or diet*) adj3 (child* or adolesc* or teen*))	(caus* or predict* or assoc* or correlat* or influence* or impact* or improve* or determin*)	(review* or cohort or longitudinal or long-term or "RCT" or "random* control* trial*" or epidemiol* or experiment*)
school adj3 read*			
early adj3 educat*			
"early child development"			
(child develop* adj3 (measure* or outcome*))			
((preschool or pre-school) adj4 (dev* or outcome))			
(cognit* adj3 (develop* or skill*) adj3 (child* or primary school*))			
(physical adj3 (develop* or skill*) adj3 (child* or primary school*))			
(language adj3 (develop* or skill*) adj3 (child* or primary school*))			
(emotion* adj3 (develop* or competen*) adj3 (child* or primary school*))			
(socioemotion* adj3 (develop* or competen*) adj3 (child* or primary school*))			
((IQ or math* or writing or "test score" or "exam score" or reading or vocabulary or literacy or numeracy or behavio* or self-regulation or achiev* or abilit*) adj3 (child* or primary school*))			

Medline (OVID) Search 12 Nov 2020:

Exposure AND Outcome = 13,622

Exposure AND outcome AND Relationship = 10381

Exposure AND outcome AND Relationship AND Studies = 5996

Limit to English language only and the last 30 years = 5734

Additional file 3: Search Strategy for Relationships between child development at school entry and adolescent health – a participatory systematic review

Exposure	Outcome	Relationship	Studies
"school readiness"	((health outcome* or health behavio* or "executive function" or anx* or depress* or "social competence" or "self-esteem" or wellbeing or obesity or weight or "mental health" or health* or diet*) adj3 (child* or adolesc* or teen*))	(caus* or predict* or assoc* or correlat* or influence* or impact* or improve* or determin*)	(review* or cohort or longitudinal or long-term or "RCT" or "random* control* trial*" or epidemiol* or experiment*)
school adj3 read*			
early adj3 educat*			
"early child development"			
(child develop* adj3 (measure* or outcome*))			
((preschool or pre-school) adj4 (dev* or outcome))			
(cognit* adj3 (develop* or skill*) adj3 (child* or primary school*))			
(physical adj3 (develop* or skill*) adj3 (child* or primary school*))			
(language adj3 (develop* or skill*) adj3 (child* or primary school*))			
(emotion* adj3 (develop* or competen*) adj3 (child* or primary school*))			
(socioemotion* adj3 (develop* or competen*) adj3 (child* or primary school*))			
((IQ or math* or writing or "test score" or "exam score" or reading or vocabulary or literacy or numeracy or behavio* or self-regulation or achiev* or abilit*) adj3 (child* or primary school*))			

Psychinfo (OVID) Search 12 Nov 2020:

Exposure AND Outcome = 18,836

Exposure AND outcome AND Relationship = 14151

Exposure AND outcome AND Relationship AND Studies = 5455

Limit to English language only and the last 30 years = 5109

Additional file 3: Search Strategy for Relationships between child development at school entry and adolescent health – a participatory systematic review

noft(health outcome* OR health behavio* OR "executive function" OR anx* OR depress* OR "social competence" OR "self-esteem" OR wellbeing OR obesity OR weight OR "mental health" OR health* OR diet*) AND noft(adolesc* OR child* OR teen*) **AND** noft("school readiness" OR school adj3 read* OR early adj3 educat* OR "early child development") **AND** noft((caus* OR predict* OR assoc* OR correlat* OR influence* OR impact* OR improve* OR determin*))

ASSIA (ProQuest) Search 12 Nov 2020:

Exposure AND Outcome = 656

Exposure AND outcome AND Relationship = 397

Limit to English language only and the last 30 years = 391

AB (school readiness or early child development or early adj3 educat*) OR AB (IQ or math* or writing or "test score" or "exam score" or reading or vocabulary or literacy or numeracy or behavio* or self-regulation or achiev* or abilit*) AND AB (child* or primary school) **AND** (adolescents or teenagers or young adults or child*) AND AB (health outcome* or health behavio* or "executive function" or anx* or depress* or "social competence" or "self-esteem" or wellbeing or obesity or weight or "mental health" or health* or diet*)) **AND** AB caus* or predict* or assoc* or correlat* or influence* or impact* or improve* or determin* **AND** (review* or cohort or longitudinal or long-term or "RCT" or "random* control* trial*" or epidemiol* or experiment*)

ERIC (EBSCO) Search 12 Nov 2020:

Exposure AND Outcome = 16330

Exposure AND outcome AND Relationship = 9582

Exposure AND outcome AND Relationship AND Studies = 5455

Limit to English language only and the last 30 years (and journal articles only) = 1307
