# Supplementary material for: Relationships between Child Development at School Entry and Adolescent Health—A Participatory Systematic Review
Source: Int J Environ Res Public Health. 2021 Nov 4;18(21):11613. doi: 10.3390/ijerph182111613 (PMC8582847; doi:10.3390/ijerph182111613)
Supplement: Supplementary file 1 [file ijerph-18-11613-s001.zip › Additional file 4 Table of excluded papers with reasons.pdf]

Additional file 4: Excluded Studies with Reasons

Authors	Year	Title	Journal	Reason
Kouros, C. D., Cummings, E. M. and Davies, P. T.	2010	Early trajectories of interparental conflict and externalizing problems as predictors of social competence in preadolescence	Development & Psychopathology	Exposure outside scope
Leech, Sharon L., Larkby, Cynthia A., Day, Richard and Day, Nancy L.	2006	Predictors and Correlates of High Levels of Depression and Anxiety Symptoms among Children at Age 10	Journal of the American Academy of Child and Adolescent Psychiatry	Exposure outside scope
Lloyd, Jennifer E. V., Li, Leah and Hertzman, Clyde	2010	Early experiences matter: Lasting effect of concentrated disadvantage on children's language and cognitive outcomes	Health & Place	Exposure outside scope
Lubans, D. R., Morgan, P. J., Cliff, D. P., Barnett, L. M. and Okely, A. D.	2010	Fundamental movement skills in children and adolescents: review of associated health benefits	Sports Medicine	Systematic review - relevant studies extracted
Lucia, V. C. and Breslau, N.	2006	Family cohesion and children's behaviour problems: a longitudinal investigation	Psychiatry Research	Exposure outside scope
Mazza, James J., Abbott, Robert D., Fleming, Charles B., Harachi, Tracy W., Cortes, Rebecca C., Park, Jisuk, Haggerty, Kevin P. and Catalano, Richard F.	2009	Early Predictors of Adolescent Depression: A 7-Year Longitudinal Study	Journal of Early Adolescence	Exposure outside scope
McCormack, Jane, Harrison, Linda J., McLeod, Sharynne and McAllister, Lindy	2011	A Nationally Representative Study of the Association between Communication Impairment at 4-5 Years and Children's Life Activities at 7-9 Years	Journal of Speech, Language, and Hearing Research	Subset of children
D'Onise, K., Lynch, J. W., Sawyer, M. G. and McDermott, R. A.	2010	Can preschool improve child health outcomes? A systematic review	Social Science & Medicine	Systematic review - relevant studies extracted
Dodge, K. A., Godwin, J. and Conduct Problems Prevention Research, Group	2013	Social-information-processing patterns mediate the impact of preventive intervention on adolescent antisocial behavior	Psychological Science	Subset of children
El Nokali, N. E., Bachman, H. J. and Votruba-Drzal, E.	2010	Parent involvement and children's academic and social development in elementary school	Child Development	Exposure outside scope
Drake, K., Belsky, J. and Fearon, R. M.	2014	From early attachment to engagement with learning in school: the role of self-regulation and persistence	Developmental Psychology	Outcome outside scope
Hedenbro, M.; Rydelius, P. A.	2019	Children's abilities to communicate with both parents in infancy were related to their social competence at the age of 15	Acta Paediatrica	Exposure outside scope
Helland, Siri Saugestad; Roysamb, Espen; Wang, Mari Vaage; Gustavson, Kristin	2018	Language difficulties and internalizing problems: Bidirectional associations from 18 months to 8 years among boys and girls	Development and Psychopathology	Outcome outside scope

Authors	Year	Title	Journal	Reason
Hendricker, Elise; Reinke, Wendy M.	2017	Conceptualizing Family Risk in a Racially/Ethnically Diverse, Low-Income Kindergarten Population	Contemporary School Psychology	Exposure outside scope
Hinkley, T.; Timperio, A.; Salmon, J.; Hesketh, K.	2017	Does Preschool Physical Activity and Electronic Media Use Predict Later Social and Emotional Skills at 6 to 8 Years? A Cohort Study	Journal of Physical Activity & Health	Exposure outside scope
Jamnik, M. R.; DiLalla, L. F.	2019	Health Outcomes Associated With Internalizing Problems in Early Childhood and Adolescence	Frontiers in Psychology	Outcome outside age range
Jaspers, M.; de Winter, A. F.; Veenstra, R.; Ormel, J.; Verhulst, F. C.; Reijneveld, S. A.	2012	Preventive child health care findings on early childhood predict peer-group social status in early adolescence	Journal of Adolescent Health	Exposure outside age range
Kantomaa, Marko T.; Stamatakis, Emmanuel; Kankaanpaa, Anna; Kaakinen, Marika; Rodriguez, Alina; Taanila, Anja; Ahonen, Timo; Jarvelin, Marjo-Riitta; Tammelin, Tuija	2013	Physical activity and obesity mediate the association between childhood motor function and adolescents' academic achievement	PNAS Proceedings of the National Academy of Sciences of the USA	Exposure and Outcome outside age range
Kim, Hyunah; Chang, Hyein	2019	Longitudinal association between children's callous-unemotional traits and social competence: Child executive function and maternal warmth as moderators	Frontiers in Psychology Vol 10 2019, ArtID 379	Exposure outside scope
Mesman, J.; Koot, H. M.	2000	Child-reported depression and anxiety in preadolescence: II. Preschool predictors	Journal of the American Academy of Child & Adolescent Psychiatry	Exposure outside age range
Miller, A. B.; Machlin, L.; McLaughlin, K. A.; Sheridan, M. A.	2020	Deprivation and psychopathology in the Fragile Families Study: A 15-year longitudinal investigation	Journal of Child Psychology & Psychiatry & Allied Disciplines	Exposure outside scope
Nantel-Vivier, Amelie; Pihl, Robert O.; Cote, Sylvana; Tremblay, Richard E.	2014	Developmental association of prosocial behaviour with aggression, anxiety and depression from infancy to preadolescence	Journal of Child Psychology and Psychiatry	Exposure outside age range
Oberer, Nicole; Gashaj, Venera; Roebers, Claudia M.	2018	Executive functions, visual-motor coordination, physical fitness and academic achievement: Longitudinal relations in typically developing children	Human Movement Science	Outcome outside scope
Piche, G.; Fitzpatrick, C.; Pagani, L. S.	2015	Associations Between Extracurricular Activity and Self-Regulation: A Longitudinal Study From 5 to 10 Years of Age	American Journal of Health Promotion	Duplicate data from same author
Piek, J. P.; Barrett, N. C.; Smith, L. M.; Rigoli, D.; Gasson, N.	2010	Do motor skills in infancy and early childhood predict anxious and depressive symptomatology at school age?	Human Movement Science	Exposure outside age range
Pihlakoski, Leena; Sourander, Andre; Aromaa, Minna; Rautava, Paivi; Helenius, Hans; Sillanpaa, Matti	2006	The continuity of psychopathology from early childhood to preadolescence: A prospective cohort study of 3-12-year-old children	European Child & Adolescent Psychiatry	Subset of children

Authors	Year	Title	Journal	Reason
Pitzer, M.; Jennen-Steinmetz, C.; Esser, G.; Schmidt, M. H.; Laucht, M.	2011	Prediction of preadolescent depressive symptoms from child temperament, maternal distress, and gender: results of a prospective, longitudinal study	Journal of Developmental & Behavioral Pediatrics	Exposure outside age range
Prior, M.; Smart, D.; Sanson, A.; Oberklaid, F.	2001	Longitudinal predictors of behavioural adjustment in pre-adolescent children	Australian & New Zealand Journal of Psychiatry	Exposure outside age range
Rende, Richard D.	1993	Longitudinal relations between temperament traits and behavioral syndromes in middle childhood	Journal of the American Academy of Child & Adolescent Psychiatry	Exposure outside age range
Sanson, Ann; Hemphill, Sheryl A.; Smart, Diana	2004	Connections between Temperament and Social Development: A Review	Social Development	Literature review but no specific data to ages of interest
Seeyave, D. M.; Coleman, S.; Appugliese, D.; Corwyn, R. F.; Bradley, R. H.; Davidson, N. S.; Kaciroti, N.; Lumeng, J. C.	2009	Ability to delay gratification at age 4 years and risk of overweight at age 11 years	Archives of Pediatrics & Adolescent Medicine	Exposure outside scope
Sourander, A.; Helstela, L.	2005	Childhood predictors of externalizing and internalizing problems in adolescence. A prospective follow-up study from age 8 to 16	European Child & Adolescent Psychiatry	Exposure and Outcome outside age range
Sulik, M. J.; Blair, C.; Mills-Koonce, R.; Berry, D.; Greenberg, M.; Family Life Project, Investigators	2015	Early Parenting and the Development of Externalizing Behavior Problems: Longitudinal Mediation Through Children's Executive Function	Child Development	Outcome outside age range
te Velde, S. J.; van Nassau, F.; Uijtdewilligen, L.; van Stralen, M. M.; Cardon, G.; De Craemer, M.; Manios, Y.; Brug, J.; Chinapaw, M. J.; ToyBox-study, group	2012	Energy balance-related behaviours associated with overweight and obesity in preschool children: a systematic review of prospective studies	Obesity Reviews	Systematic review - relevant studies extracted
Tearne, J. E.; Allen, K. L.; Herbison, C. E.; Lawrence, D.; Whitehouse, A. J.; Sawyer, M. G.; Robinson, M.	2015	The association between prenatal environment and children's mental health trajectories from 2 to 14 years	European Child & Adolescent Psychiatry	Exposure outside age range
Tinsley, B. J.	1992	Multiple influences on the acquisition and socialization of children's health attitudes and behavior: an integrative review	Child Development	Theory paper but limited relevance to the RQ
Trentacosta, C. J.; Fine, S. E.	2010	Emotion Knowledge, Social Competence, and Behavior Problems in Childhood and Adolescence: A Meta-Analytic Review	Social Development	Exposure and Outcome outside age range
Troller-Renfree, S. V.; Buzzell, G. A.; Bowers, M. E.; Salo, V. C.; Forman-Alberti, A.; Smith, E.; Papp, L. J.; McDermott, J. M.; Pine, D. S.; Henderson, H. A.; Fox, N. A.	2019	Development of inhibitory control during childhood and its relations to early temperament and later social anxiety: unique insights provided by latent growth modeling and signal detection theory	Journal of Child Psychology & Psychiatry & Allied Disciplines	Exposure outside age range

Authors	Year	Title	Journal	Reason
Uszynska-Jarmoc, Janina	2007	Self-Esteem and Different Forms of Thinking in Seven and Nine Year Olds	Early Child Development and Care	Exposure outside scope
Vazsonyi, A. T.; Huang, L.	2010	Where self-control comes from: on the development of self-control and its relationship to deviance over time	Developmental Psychology	Exposure outside scope
Visser, L.; de Winter, A. F.; Vollebergh, W. A.; Verhulst, F. C.; Reijneveld, S. A.	2015	Do child's psychosocial functioning, and parent and family characteristics predict early alcohol use? The TRAILS Study	European Journal of Public Health	Exposure outside age range
Vlahov, Eric; Baghurst, Timothy M.; Mwavita, Mwarumba	2014	Preschool motor development predicting high school health-related physical fitness: A prospective study	Perceptual and Motor Skills	Outcome outside age range
Wang, Cen; Williams, Kate E.; Shahaeian, Ameneh; Harrison, Linda J.	2018	Early Predictors of Escalating Internalizing Problems across Middle Childhood	School Psychology Quarterly	Subset of children
Wang, Yiji; Dix, Theodore	2017	Mothers' Depressive Symptoms in Infancy and Children's Adjustment in Grade School: The Role of Children's Sustained Attention and Executive Function	Developmental Psychology	Exposure outside scope
Warren, A. S.; Goldsmith, K. A.; Rimes, K. A.	2019	Childhood gender-typed behavior and emotional or peer problems: a prospective birth-cohort study	Journal of Child Psychology & Psychiatry & Allied Disciplines	Exposure outside scope
Watts, Tyler W.; Gandhi, Jill; Ibrahim, Deanna A.; Masucci, Michael D.; Raver, C.	2018	The Chicago School Readiness Project: Examining the long-term impacts of an early childhood intervention	PLoS ONE	Exposure outside scope
Weeks, Murray; Cairney, John; Wild, T.; Ploubidis, George B.; Naicker, Kiyuri; Colman, Ian	2014	Early-life predictors of internalizing symptom trajectories in Canadian children	Depression and Anxiety	Exposure outside scope
Weeks, Murray; Ploubidis, George B.; Cairney, John; Wild, T.; Naicker, Kiyuri; Colman, Ian	2016	Developmental pathways linking childhood and adolescent internalizing, externalizing, academic competence, and adolescent depression	Journal of Adolescence	Outcome outside age range
Wirt, T.; Hundsdorfer, V.; Schreiber, A.; Kesztyus, D.; Steinacker, J. M.; Komm mit in das gesunde Boot - Grundschule" - Research, Group	2014	Associations between inhibitory control and body weight in German primary school children	Eating Behaviors	Study design
Woodward, L. J.; Lu, Z.; Morris, A. R.; Healey, D. M.	2017	Preschool self regulation predicts later mental health and educational achievement in very preterm and typically developing children	Clinical Neuropsychologist	Exposure outside age range
Xue, Y.; Leventhal, T.; Brooks-Gunn, J.; Earls, F. J.	2005	Neighborhood residence and mental health problems of 5- to 11-year-olds	Archives of General Psychiatry	Exposure outside scope
Sabol, T. J.; Pianta, R. C.	2011	Patterns of School Readiness Forecast Achievement and Socioemotional Development at the End of Elementary School	Child Development	Study design
