# Supplementary material for: Relationships between Child Development at School Entry and Adolescent Health—A Participatory Systematic Review
Source: Int J Environ Res Public Health. 2021 Nov 4;18(21):11613. doi: 10.3390/ijerph182111613 (PMC8582847; doi:10.3390/ijerph182111613)
Supplement: Supplementary file 1 [file ijerph-18-11613-s001.zip › Additional file 5 Data Extraction Form.pdf]

Additional file 5: Data Extraction Form

<table>
<tr>
<td>
Authors:

Country:

Study objectives:

Study design :
<table>
<tr><td>Descriptive</td><td></td></tr>
<tr><td>RCT</td><td></td></tr>
<tr><td>Cohort</td><td></td></tr>
<tr><td>Sys. review</td><td></td></tr>
<tr><td>Longitudinal Analysis</td><td>X</td></tr>
<tr><td>Qualitative</td><td></td></tr>
<tr><td colspan="2">Other:</td></tr>
</table>

Study population: (including gender split and year recruited)

Contextual factors:

Data collection methods/sources:
</td>
<td>
Exposure, age and measurement instrument:

Mediating Variables:

Outcomes, age and measurement instrument:

Association and effect size:

Co-variates/Confounders:

Mechanisms between exposure and outcome:

Moderators of the relationship between exposure and outcome:

Summary of Findings:
</td>
<td>
Main author conclusions:

Strengths:

Weaknesses:
</td>
</tr>
</table>
