# Supplementary material for: Relationships between Child Development at School Entry and Adolescent Health—A Participatory Systematic Review
Source: Int J Environ Res Public Health. 2021 Nov 4;18(21):11613. doi: 10.3390/ijerph182111613 (PMC8582847; doi:10.3390/ijerph182111613)
Supplement: Supplementary file 1 [file ijerph-18-11613-s001.zip › Additional file 6 QA template.pdf]

Additional file 6: Quality Assessment Form

STUDY ID	Main Author and date	EXTRACTED BY		EXTRACTION DATE	DD	MM	YY

METHODOLOGICAL QUALITY APPRAISAL (adapted from previously used assessments in systematic reviews[1,2]

Relationships between child development at school entry and adolescent health: A participatory systematic review

Quality Assessment Criteria	I/V/P	+/-/?
Study Population		
1. Adequate* description of sampling frame†	I	
2. Participation rate at baseline at least 80%, or evidence that the non-response is not selective	V	
3. Adequate* description of baseline study sample†	I	
Study Attrition		
4. Provision of the number of participants at each follow up	I	
5. Provision of information on follow-up duration	I	
6. Response at follow up was at least 70% of the number of participants at baseline	V	
7. Not selective non-response during follow-up assessments‡	V/P	
Data collection		
8. Measurement of exposures using objective or valid and reliable measures§	V	
9. Measurement of outcomes using objective or valid and reliable measures§	V	
Data Analysis		
10. Appropriate statistical model used	V/P	
11. The majority of known confounders in the model	V/P	
12. Presentation of point estimates and measures of variability (confidence intervals)	I	
**Quality Ranking:** Low , Moderate , High		

* Adequate = sufficient information to be able to repeat the study.
† '+' is given only if adequate information is given in the text on all items.
‡ '+' is given only if non-selective dropout study sample does not significantly differ from study population on key characteristics or results are adjusted for selective non-response (via appropriate missing data imputation procedures).
§ (+ = objective or valid/reliable measurements for the relevant measures – author stated validity and reliability and reference)
^ '+' is given if gender, measure of SES such as family income/Maternal education, maternal mental health and baseline outcome measure at age of exposure (for cross domain only). If a major confounder is missing this is a red flag item and the study is rated low.
I/V/P criterion: Informativeness (relates to clarity of study description), Validity and Precision (relate to risk of bias).

***Notes: Assigning the quality ranking**: V and P criteria should strongly guide the overall quality ranking and carry a greater weight than I. For example all V and P with minimal I would indicate high quality, whereas all I and minimal V and P would indicate low quality*
Studies with limited reporting of method or results, those with minimal or no confounders and those with high attrition and no adjustment made for non-response or missing data are judged to have a high risk of bias and rated to be of low methodological quality. Those with adequate reporting, most measurement instruments valid, some confounders and adjustments made for non-response and missing data should be rated as moderate. Those with clear reporting, all measurement instruments valid, a comprehensive list of confounders and adjustments made for non-response and missing data are considered to have a low risk of bias/high quality.

---

[1] Hoogendoorn WE, van Poppel MN, Bongers PM *et al*. Systematic review of psychosocial factors at work and private life as risk factors for back pain. *Spine (Phila Pa 1976)* 2000; 25: 2114–2125.
[2] te Velde, S.J., van Nassau, F., Uijtdewilligen, L *et al*. Energy balance-related behaviours associated with overweight and obesity in preschool children: a systematic review of prospective studies. *Obesity Reviews*, 2012, 13: 56-74.
