# Supplementary material for: Relationships between Child Development at School Entry and Adolescent Health—A Participatory Systematic Review
Source: Int J Environ Res Public Health. 2021 Nov 4;18(21):11613. doi: 10.3390/ijerph182111613 (PMC8582847; doi:10.3390/ijerph182111613)
Supplement: Supplementary file 1 [file ijerph-18-11613-s001.zip › Additional File 8 table of study characteristics.pdf]

Additional File 8: Table of study characteristics

Author (Year)	Study Design	Country	Participants	Exposure (measure of development) and age	Exposure Measurement Instrument	Outcome and age	Outcome Measurement Instrument	Results	Mechanism - in study (normal font) or *theorized (italics)*	Factors which moderate the association
Ashford et al 2008	Longitudinal	Holland	294 children	Behaviour internalizing and externalizing - age 4	Child Behaviour Checklist (CBCL) - parent and teacher rated.	Internalizing behaviours - age 11	CBCL – parent and teacher report.	Externalizing and internalizing problems both associated with internalizing problems with an IRR of 2.81 (p<0.001) and 3.83 (p<0.001) respectively. Four risk indicators (low SES, parenting stress at age 4-5, internalizing at age 4-5 and family psychopathology age 2-3) associated with internalizing behaviours and together resulted in a total AF of 57%, implying that with this set of risk indicators, 57% of the future cases of internalizing problems can be identified.	-	Low SES and parenting stress also predict the outcome.
Berthelsen et al 2017	Longitudinal	Australia	4819 Children from the Growing up in Australia: The Longitudinal Study of Australian Children.	Child Behaviour at age 4-5 and early ecological risk factors SEP, MMH, Parenting anger, parenting warmth, parenting consistency.	Child behaviour risk index measured as the sum of scores: sleep (parent report), emotional dysregulation (parent report) and inattention/ hyperactivity symptoms (mother rated)	Executive Function (age 14-15).	A composite score from three Cogstate computerised tasks for assessing cognition and measured visual attention, visual working memory and spatial problem solving.	Negative association between child behaviour risk index and executive function (β = -0.09). Full model (all early ecological risk factors): Total effects on EF for child behaviour risk (β=-0.07) and attentional regulation (β = 0.13) p<0.001 for both. Attentional regulation at 4–5 years (β = 0.10) and approaches to learning at 6–7 years (β = 0.18) were both directly associated with executive function at 14–15 years.	Attentional regulation and approaches to learning mediated the relationship between behavioural risk and EF.	Low SEP is an additional risk factor for lower EF. Poorer maternal mental health and parenting also associated indirectly with EF via effect on children's self-regulation skills.

Author (Year)	Study Design	Country	Participants	Exposure (measure of development) and age	Exposure Measurement Instrument	Outcome and age	Outcome Measurement Instrument	Results	Mechanism - in study (normal font) or *theorized* (*italics*)	Factors which moderate the association
Bornstein et al 2010	Longitudinal	US east coast	118 European American families with children aged 4	Social competence at age 4	Social competence as a construct, of: the peer acceptance subscale of the Pictorial Scale of Perceived Competence and Social Acceptance Preschool Form, the Friendship Interview, and the socialization domain of the Vineland Adaptive Behavior Scales (VABS).	Internalizing and externalizing behaviours at age 10 and 14	At age 10 years - the CBCL and Teacher Report Form. At age 14 years - the CBCL and Youth Self-Report.	Social competence predicted:<br>• Externalising behaviours at age 14 (β =-0.14, $p<0.05$), mediated by internalizing and externalizing behaviours at age 10<br>• Internalizing behaviour at age 14 (β= -0.09, $p < 0.05$) mediated by internalizing behaviours at age 10.	Social competence at age 4 predicts externalizing and internalizing behaviours at age 14. For externalizing behaviour this relationship is mediated by internalising and externalising behaviour at age 10. For internalizing behaviour the relationship is mediated by internalizing behaviours only.	
Bornstein et al 2013	Longitudinal	US east coast	224 European American families - two studies (Study 2 extracted only - 139 children)	Language – communication skills - at age 4	Two verbal subtests of the Wechsler Preschool and Primary Scale of Intelligence—Revised and the VABS	Internalizing and externalizing behaviours at age 10 and 14	At age 10 years - the CBCL and Teacher Report Form. At age 14 years - the CBCL and Youth Self-Report.	Language skills predicted age 14-year internalizing behaviour problems (β= –0.12, $p < .005$), mediated by 10-year internalizing behaviour problems. Language skills did not predict externalizing behaviour problems.	Age 10-year internalizing behaviour problems mediate the relationship between language skills at age 4 and internalizing problems at age 14.	

Author (Year)	Study Design	Country	Participants	Exposure (measure of development) and age	Exposure Measurement Instrument	Outcome and age	Outcome Measurement Instrument	Results	Mechanism - in study (normal font) or *theorized (italics)*	Factors which moderate the association
Derks et al 2019	Cohort	The Netherlands	Three population cohort studies: only 1 relevant study: Generation R study, 3794 children aged 6-10 in The Netherlands	Aggressive behaviour - at ages 5-7, 10 and 14	CBCL - mother rated	BMI and body composition (fat mass and fat free mass) - at ages 6 and 10	BMI - the Dutch national reference in the Growth Analyser program. FM and FFM - dual-energy x-ray absorptiometry scanner	Aggressive behaviour at age 6 years was associated with higher BMI at age 10 years (β = 0.02, 95% CI: 0.00 to 0.04) and higher FMI at age 10 years β = 0.03, 95% CI: 0.01 to 0.05). No association found in the opposite direction.	*Eating behaviour (self-regulation) and aggressive behaviour are regulated by the same neurotransmitter pathways. Children with aggressive behaviour may also have deficits in self-regulation leading to over eating and obesity. Other mechanisms proposed: Aggressive behaviour and BMI share genetic vulnerabilities and the mechanism of parenting with parents using food to cope with challenging behaviour of children.*	
Duchesne et al 2010	Longitudinal	Canada	2000 children from the Quebec Longitudinal Study of Kindergarten Children	Behaviour - hyperactivity, inattention, aggressiveness and prosociality - age 6<br><br>Maternal warmth and maternal control also studied.	Social Behaviour Questionnaire (SBQ) - teacher rated	Trajectory of anxiety at age 11-12	Rated annually from kindergarten to Grade 6 using the Anxiety Scale from the SBQ – teacher report. Children put into trajectory of anxiety.	Children grouped into trajectory of anxiety. Probability of being in the 'high anxiety' group was greater for children facing adversity (odds ratio = 3.46, p < .001) and who were inattentive in the classroom (odds ratio = 6.24, p < .001). Probability of belonging to this group was lower for children who were socially competent (odds ratio = 0.66, p < .05). Children whose mothers	-	Hyperactive children less likely to be anxious in grade 6 if there was a warm mother/child relationship – protective mechanism at play.<br>Maternal discipline (rules and efforts to control child

**Author (Year)**	**Study Design**	**Country**	**Participants**	**Exposure (measure of development) and age**	**Exposure Measurement Instrument**	**Outcome and age**	**Outcome Measurement Instrument**	**Results**	**Mechanism - in study (normal font) or *theorized (italics)***	**Factors which moderate the association**
								tended to apply rules and control their behaviours had a greater probability of belonging to the high group (odds ratio = 1.07, p < .05). The probability of belonging to the high group was higher for hyperactive children interacting with mothers who showed little affective warmth.		behaviours) associated with higher anxiety.
Fine et al 2003	Longitudinal	US	154 children from economically disadvantaged families	Emotional knowledge, internalizing and ext behaviours age 7	Emotion knowledge – composite score from two tasks:<br>1. Emotional labelling – ability to label facial expressions<br>2. Emotion situation knowledge – ability to label a protagonist in a story<br>Internalizing and externalizing behaviours – CBCL (teacher report)	Internalizing behaviours age 11	Child self-report aggregate of the following measures:<br>Depression - Children's Depression Inventory (CDI)<br>Anxiety - The State-Trait Anxiety Inventory<br>Loneliness. The Loneliness Scale<br>Negative emotions – Differential emotions scale	Teacher reported externalizing behaviours at age 7 predicted child anxiety self-report at age 11 (β = .26, p < .01). Internalizing behaviours did not.<br>Emotion knowledge at age 7 significantly predicted anxiety self-reports at age 11 (β = −.23, p < .05).	*In mid-childhood when connections between emotions and cognition are developing, poor emotional knowledge may lead to negative emotional patterns leading to internalizing problems. .*<br>*Indirect mechanisms may include peer relations and social behaviour.*	

Author (Year)	Study Design	Country	Participants	Exposure (measure of development) and age	Exposure Measurement Instrument	Outcome and age	Outcome Measurement Instrument	Results	Mechanism - in study (normal font) or *theorized (italics)*	Factors which moderate the association
Glaser et al 2011	Longitudinal	UK	5250 children from the ALSPAC study, UK	IQ age 8	Wechsler Intelligence Scale for Children	Depression symptoms - age 11, 13, 14 and 17	Self-reported depressive symptoms were measured with the 13-item Short Mood and Feelings Questionnaire (SMFQ) Moderator: Pubertal stage at 11, 13 and 14 years was measured using a five-point rating scale	An increase in 1 s.d. of baseline IQ was associated with a 7% decrease in depression symptoms [SCR=0.93, 95% (CI) 0.92-0.95]. This association changed direction at ages 13 and 14 years such that an increase in 1 s.d. of baseline IQ was associated with a 4% (SCR=1.04, 95% CI 1.02-1.06) and 3% increase (SCR=1.03, 95% CI 1.02-1.05) in depression symptoms, respectively	*The authors speculate that the reappearance of the protective effect of higher childhood IQ in early adulthood, as observed for females at age 17 years, could reflect the end of pubertal development as, for example, biological stress-management systems improve during puberty.*	Association varies depending on pubertal stage - mimics the relationship by age but weaker for males than females by age 17.
Gregory et al 2020	Longitudinal	Australia	3906 children - mainly caucasian	School readiness across 5 domains (physical, social, emotional, language and cognitive, communication and general knowledge) - Age 5	Australian version of the Early Development Instrument – teacher rated. Children scored as vulnerable, at risk or on-track.	Age 11: four aspects of student wellbeing (life satisfaction, optimism, sadness and worries)	Middle Years Development Instrument - child self-report	Children classed as vulnerable in physical, social and emotional development had lower levels of life satisfaction and optimism and higher levels of sadness and worries compared to children classed as at risk. For the language and cognitive, communication and general knowledge domains the association was with sadness and worries only (lower levels of development associated with more worries). (For all $p<0.05$ or < 0.01)		In most cases, effect estimates were attenuated after adjusting for child and family level confounders. (SES, gender, Aboriginal and/or Torres Strait Islander status, language background other than English (LBOTE), parental education.

Author (Year)	Study Design	Country	Participants	Exposure (measure of development) and age	Exposure Measurement Instrument	Outcome and age	Outcome Measurement Instrument	Results	Mechanism - in study (normal font) or *theorized (italics*)	Factors which moderate the association
Hay et al 2003	Longitudinal	UK	134 children age 4 from two urban communities in London	Co-operation (one form of prosocial behaviour) at age 4	Tester's rating of cooperativeness during the cognitive test (Tester's Rating of Children's Behaviour) and an observational measure of cooperation with the mother during the Etch-A-Sketch task.	Internalizing and externalizing behaviour problems – at age 11	SDQ and CAPA (Child and Adolescent Psychiatric Assessment).	Children who were more cooperative with their mothers at age 4 had fewer externalizing problems at age 11, r(108)5 – 30, p<.002. There was no association between cooperation at age 4 and internalizing problems at age 11	*Authors suggest that active cooperation reflects emotionally regulated, socially competent engagement with the social environment may set the child on a course of successful interactions that promote general psychological adjustment.*	Maternal depression decreased prosocial behaviour in the eyes of adults, but children of depressed mothers saw themselves to be prosocial.
Hooper et al 2003	Longitudinal	US	74 African American Children	Language - receptive and expressive language, receptive vocab and working memory - age 5 and 7-8 (kindergarten and second grade)	Receptive and expressive language - The Clinical Evaluation of Language Fundamentals.<br>Receptive vocab (Peabody test) and Working memory (Competing Language Processing Task)	Behaviour problems – externalizing problems (conduct and hyperactivity) - kindergarten, first, second, and third grade	Teachers completed assessments of the children's behavior i using a standardized scale of behavior - Conners' Teacher Rating Scale-Revised	Language and conduct problems: By the time the children reached 9 years of age, the relationship between receptive language and Conduct Problems was significant, β = –0.47, p < .01<br><br>Language and hyperactivity: None of the three language measures was found to be a significant predictor of Hyperactivity at any age studied.	-	

**Author (Year)**	**Study Design**	**Country**	**Participants**	**Exposure (measure of development) and age**	**Exposure Measurement Instrument**	**Outcome and age**	**Outcome Measurement Instrument**	**Results**	**Mechanism - in study (normal font) or *theorized (italics)***	**Factors which moderate the association**
Howard et al 2018	Cohort	Australia	4983 kindergarten children from the Longitudinal Study of Australian Children (LSAC)	Self-regulation - age 4-5 and 6-7	Self-regulation problems were indexedby combining parent-, teacher-, and interviewer-report ratings of children's self-regulatory behaviours	Academic and weight, mental health, substance use, crime, self-harm and suicidal ideation - age 15	• Academic achievement - children's total scores on the Year 9 National Assessment Program - Literacy and Numeracy<br>• Mental health problems were measured in a private face-to-face interview with the parent/carer who knew the adolescent best<br>• Overweight and obesity status was calculated using height and weight which were converted to body mass index scores and used to calculate overweight and obese categories	• Self-regulation problems at 4 to 5 years and at 6 to 7 years were significantly associated with each adolescent outcome. A 1-unit increase in self-regulation problems at either age was associated with one-fifth of an SD reduction in reading and numeracy scores 1 decade later.<br>• Self-regulation problems were associated with a 1.2- to 1.4-times increase in the risk of being an overweight or obese adolescent. Change in early self-regulation (reduced problems) had no effect on the association.<br>• 1 SD increase in self-regulation problems was associated, in adolescence, with a more than a 2-times increase in the risk of self-harm ideation and behaviour, suicidal ideation, and school truancy; almost a 2-times increase in mental health problems, smoking, and violent and property crime; and more than a 1.5-times risk of alcohol use. Change in self-regulation: the association with earlier self-regulation problems was no longer found.	*Social Cognitive Theory*	

Author (Year)	Study Design	Country	Participants	Exposure (measure of development) and age	Exposure Measurement Instrument	Outcome and age	Outcome Measurement Instrument	Results	Mechanism - in study (normal font) or *theorized (italics)*	Factors which moderate the association
Howes et al 2000	Longitudinal	US	307 pre-school children in a longitudinal study	Preschool social—emotional climate, Peer play, Behaviour problems, Teacher-child relationship quality - Age 4	Preschool social—emotional climate - average of children's scores on selected measures within a classroom. Peer play – peer play scale Behaviour problems – classroom behaviour inventory (CBI) Teacher perceptions of their relationship with the child were assessed with the Pianta Student Teacher Relationship Scale	Social competence - Behaviour with peers at age 8	Teacher reports using the Cassidy and Asher Teacher Assessment of Social behaviour Questionnaire	Children's second grade social competence with peers ratings could be predicted by preschool classroom social-emotional climate, four year-old behaviour problems and child-teacher relationship quality.	*Attachment theory and attention to the social context of the classroom*	
Jaspers et al 2010	Longitudinal (retrospective)	Holland	2139 children aged 11 in the TRacking Adolescents'	Behavioural features at age 4 - 'sleeping, eating, and	Assessed by Preventative Child Healthcare	Behavioural and emotional problems	CBCL - parent completed.	Behaviour problems and attention problems predicted externalizing problems with an adj OR 2.3 (1.2-4.2) and 2.1 (1.3-		Low level of education of the father, and being male were identified

Author (Year)	Study Design	Country	Participants	Exposure (measure of development) and age	Exposure Measurement Instrument	Outcome and age	Outcome Measurement Instrument	Results	Mechanism - in study (normal font) or *theorized (italics)*	Factors which moderate the association
			Individual Lives Survey (TRAILS)	enuresis problems' and 'emotional and behaviour problems'	professionals.	at age 10 to 12		3.3) respectively. The behaviours did not predict internalizing problems.		as significant independent determinants of clinical externalizing problems. Sleep problems (at age 4), maternal smoking during pregnancy, being male were independent determinants of clinical internalizing problems
Lecompte et al 2014	Longitudinal	Canada	68 children from SE diverse parts of Montreal	Emotional wellbeing - Child-parent attachment at age 3-4	Lab based separation reunion procedure	Anxiety and depressive symptoms and self-esteem (age 11-12)	Dominic Interactive Questionnaire -computerised self-report measure of common mental health disorders in childhood. Self-esteem - self-perception profile for children - self-report	Disorganized attachment compared to secure:<br>• Higher Anxiety Symptoms: $\beta = -2.88$, $p < .05$<br>• Higher Depressive symptoms: $\beta = -3.27$, $p < .01$<br>• Lower Self-esteem $\beta = 2.61$, $p < .05$	Disorganized attachment associated with higher anxiety and depression in pre-adolescence. The effect was partially mediated by self-esteem for depression but not anxiety.	

Author (Year)	Study Design	Country	Participants	Exposure (measure of development) and age	Exposure Measurement Instrument	Outcome and age	Outcome Measurement Instrument	Results	Mechanism - in study (normal font) or *theorized (italics)*	Factors which moderate the association
Lee et al 2017	Longitudinal	US	762 Children from the Fragile Family and Wellbeing Study	Behaviour internalizing and externalizing - age 5	CBCL - primary caregiver completed	Behaviour internalizing and externalizing - age 9	CBCL - primary caregiver completed	• Internalizing behaviour problems at age 5 were more likely to show higher internalizing behaviour problems at age 9, β = .44, p < .001.<br>• Externalizing behavior problems at age were more likely to show higher externalizing behavior problems at age 9. (β = .45, p < .001)<br>• Fathers' greater positive engagement reduced the association between poverty and internalizing behavior problems at age 9, β = –.08, p = .014 and for externalizing problems (β = –.08, p = .008)<br>• For families experiencing greater poverty fathers' positive engagement was associated with a reduction in the continuity of internalizing problems from 5 to 9 years of age.		Poverty: Greater internalizing and externalizing probs were seen for children living in poverty but this was buffered by father's positive engagement. In addition continuity of internalizing problems is weakened by father's positive engagement for children living below the poverty line- *via secure attachment and development of emotional and behavioural regulation skills.*
Louise at al 2012	Longitudinal	Western Australia	2900 children of Women in the Western Australian Pregnancy Cohort (Raine) Study -	Behaviour - aggressive - age 5, 8, 10 and 14	CBCL, youth self-report at age 14 and teacher report at age 10 and 14	Weight at age 5, 8,10 and 14	Weight - Wedderburn digital chair scale Height was measured using Holtain Stadiometer. BMI was calculated as weight	• Girls with higher aggressive behaviour scores throughout childhood had a higher rate of change of their BMI. This association persisted after adjusting for race and family income (βage × agg score = 0.005 kg/m2; 95% CI: 0.002, 0.008; P = 0.001).	*Aggression and BMI: mechanism proposed is that aggression and BMI may be linked by a common environmental (e.g. low self-esteem) or biological factor (e.g. leptin)*<br>*Aggression and BP*	

Author (Year)	Study Design	Country	Participants	Exposure (measure of development) and age	Exposure Measurement Instrument	Outcome and age	Outcome Measurement Instrument	Results	Mechanism - in study (normal font) or *theorized (italics)*	Factors which moderate the association
			majority caucasian				(kg)/height2 (m2). Blood pressure was measured using a Dinamap electronic blood pressure recorder.	• Boys with higher aggressive behaviour scores throughout childhood had a lower rate of change in their systolic blood pressure This association persisted after adjusting for BMI (βage × agg score = −0.021 mmHg/year; 95% CI: −0.037,−0.005; P = 0.010).<br>• No associations were detected between aggressive behaviour scores and BMI trajectories in boys, systolic blood pressure trajectories in girls or diastolic blood pressure trajectories in either boys or girls	*Physical aggressive behaviours such as arguing, attacking and fighting are assessed by CBCL, YSR and TRF in this study. As such, suppressed aggression maybe associated with hypertension, whereas physical expression of aggressive behaviour may be associated with lower systolic blood pressure*	
McKenzie et al 2002	Longitudinal	USA	207 Mexican American and Anglo-American Children in the San Diego Study of Children's Activity and Nutrition (SCAN) project	Fundamental movement skills - Balance, agility, eye-hand coordination - age 4,5 and 6	Movement skill tests in the child's home	Physical Activity - age 12	Trained assessors administered the 7-day Physical Activity Recall (PAR) in the child's home on two occasions, approximately 6 months apart.	Movement skill performances of the children at ages 4-6 years did not predict physical activity levels at the age of 12 years.	*The authors state that physical activity and movement skills are modifiable through interventions, including school PE and sports. The physical education and sport instruction the children received after the age of 6 years may have reduced the tracking of movement performances and the relationship of early skills to later*	

**Author (Year)**	**Study Design**	**Country**	**Participants**	**Exposure (measure of development) and age**	**Exposure Measurement Instrument**	**Outcome and age**	**Outcome Measurement Instrument**	**Results**	**Mechanism - in study (normal font) or *theorized (italics*)**	**Factors which moderate the association**
									physical activity engagement.	
Meagher et al 2009	Longitudinal	USA	56 children from childcare centres in New England - majority socially disadvantaged	Socio-emotional behaviours observed in pre-school – age 4	Externalizing and internalizing symptoms from the CBCL – teacher report Observed negative effect by research assistants	Depression symptoms - age 8	Child depression inventory - self-report	Internalizing and externalizing symptoms not predictive of depression symptoms (internalizing). Rule breaking and observed negative affect in preschool were stronger predictors of later depressive symptoms in girls than in boys	*The authors suggest that the findings that rule breaing and negative affect predicted later depressive symptoms suggest that behaviors that place children at-risk for social isolation and adult disapproval may be a pathway to depressive symptoms*	Sex - Rule breaking at 4 was associated with depression in girls at age 8
Nelson et al 2018	Longitudinal	US	280 pre-school children and their mothers in a small Midwestern city - oversampled foe socioeconomic risk	Executive control and Foundatinal Cognitive Abilities at age 5	9-tasks administered to each child during individual sessions in the laboratory. The tasks were designed to cover the major areas that make	Depression and Anxiety symptoms - Age 9-10.	Child Depression Inventory – child self-report Anxiety symptoms - Revised Child Manifest Anxiety Scale, - child self-report Externalizing symptoms -	• Preschool EC significantly predicted depression symptoms, with poorer EC associated with greater depression symptoms, $b = -2.59$, $ß = -0.25$, $SE = 0.10$, $t = -2.54$, $p = 0.011$.<br>• EC also significantly predicted anxiety symptoms, such that children with poorer EC had greater anxiety symptoms, $b = -3.66$, $ß = -0.28$,	*One potential pathway could be through the "impaired disengagement hypothesis"whereby problems with attentional control lead to disengagement and low mood. The role of EC may be critical in moderating*	Sex – girls more anxiety than boys

Author (Year)	Study Design	Country	Participants	Exposure (measure of development) and age	Exposure Measurement Instrument	Outcome and age	Outcome Measurement Instrument	Results	Mechanism - in study (normal font) or *theorized* (*italics*)	Factors which moderate the association
					up EC, including working memory, inhibitory control, and flexible shifting and FCA via the Woodcock-Johnson-III Brief Intellectual Assessment		parents completed the ODD and ADHD-Hyperactivity subscales of the Conners 3rd Edition Parent Ratings Scale	SE = 0.09, t = −2.98, p = 0.003.<br>• FCA did not significantly predict depression or anxiety symptoms<br>• Preschool EC did not significantly predict at-risk/clinical levels of depression but did significantly predict anxiety (log odds = −0.92, p = 0.048), with lower EC scores associated with greater likelihood of at-risk/clinical levels of anxiety symptoms.<br>• FCA did not significantly predict at-risk/clinical levels of depression or anxiety symptoms	*experiences of negative emotions.*	
Pagani et al 2014	Longitudinal	Canada	1145 children from the Quebec Longitudinal Study of Child Development - largely middle class	Cognitive skills, Attention skills - age 5-6	Cognitive skills: Number Knowledge Test (administered to children by trained examiners), Receptive vocabulary skills (The Peabody Picture Vocabulary Test administered to children	Wellbeing - age 10	Health-related behaviours – parent report on child food intake (soft drinks, sweet snacks, fruit and veg and dairy) and physical effort during free time<br>Academic achievement - Children completed the Canadian Achievement	Receptive vocabulary in kindergarten exclusively predicted fourth-grade dietary habits. Unstandardized coefficients predicted decreases in sweet snack intake (β = −.009, 95% confidence interval [CI] = −.011 to −.006) and dairy product intake (β = .009, 95% CI = .005 to .013). Conversely, higher kindergarten math skills predicted increases in activities requiring physical effort (β = .030, 95% CI = .011 to .056). Although vocabulary and attention	*The authors state that 'school readiness represents a means to improve self-efficacy, which mobilizes motivation and resilience in achieving cognitive skills for initiating autonomous healthy behavior and its maintenance'. This implies that school readiness supports better critical thinking about lifestyle habits and behaviors.*	

Author (Year)	Study Design	Country	Participants	Exposure (measure of development) and age	Exposure Measurement Instrument	Outcome and age	Outcome Measurement Instrument	Results	Mechanism - in study (normal font) or *theorized (italics)*	Factors which moderate the association
					by trained examiners), Attention skills – teacher assessed from the Social Behaviour Questionnaire (SBQ)		Test of mathematics with a trained examiner. Psychosocial adjustment - Teachers report and child self-report on child classroom behaviour – SBQ.	skills were found important, kindergarten math skills were stronger and more consistent predictors of later academic outcomes.		
Pedersen et al 2007	Longitudinal	Canada	551 French-Canadian children from a small community in North West Quebec.	Behaviour - anxiety/social withdrawal and disruptive behaviour - Age 6	Social Behaviour Questionnaire (SBQ) - mother and teacher rated	Peer rejection (age 8 to 11), Friendedness (at age 8 to 11) Depressive symptoms Loneliness Delinquency - at Age 13	• Peer rejection Children's sociometric status was assessed through peer nominations. •Friendedness Children were also asked to nominate up to four best friends in the classroom • Depressive symptoms - CDI - child report • Loneliness–social dissatisfaction -self-report measure	Early behaviour was associated with both middle-childhood peer processes. Early disruptiveness was positively related to peer rejection at ages 8–9 and 10–11 (ages 8–9: estimate = .36, p < .001; ages 10–11: estimate = .17, p < .01) and negatively related to friendedness at ages 8–9 (estimate =–.16, p < .01). Anxiety–social withdrawal, however, was only associated with the number of friends at ages 8–9 (estimate =–.17, p < .01). • indirect pathway from disruptiveness to depressive symptoms via peer rejection at ages 8–9 and friendedness at ages 10–11 was statistically	Peer rejection and Friendedness: *In relation to peer rejection not having a mediating role linking disruptive behaviour with externalizing outcomes: there are theoretical models which suggest that personal characteristics are the sole predictors of later antisocial behaviours, with peer experiences playing only an incidental role in this process*	Boys tended to be more disruptive and report more early-adolescent delinquency. Girls reported more early-adolescent depressive symptoms. Few sex differences, however, were observed in the middle-childhood peer processes after adjusting for early behavior. Lower occupational status was related to

Author (Year)	Study Design	Country	Participants	Exposure (measure of development) and age	Exposure Measurement Instrument	Outcome and age	Outcome Measurement Instrument	Results	Mechanism - in study (normal font) or *theorized (italics)*	Factors which moderate the association
							developed by Asher et al 1984<br>• Delinquency Involvement - assessed Self-Reported Delinquency Questionnaire (SRDQ)	significant (estimate = .01, p < .05).<br>• indirect path emerged from disruptiveness to loneliness via peer rejection at ages 8–9 and 10–11 (estimate = .03, p < .01).<br>• No mediation process found for delinquency but early disruptiveness significantly predicted adolescent delinquency (estimate = .19, p < .01).		greater childhood disruptiveness and more early-adolescent depression, loneliness, and delinquency.
Piche et al 2012	Longitudinal	Canada	966 from the Quebec Longitudinal Study of Child Development	Self-regulatory skills: classroom engagement and behavioural regulation (emotional distress, physical aggression, impulsivity) - Age 6	Classroom engagement (teacher rated) and Behavioural regulation using the SBQ (teacher rated) –	Child Sports Participation and BMI - Age 10	Parents reported on their child's weekly involvement in structured sports outside of school during the past school year.<br>BMI was derived from direct height and weight measures made by trained, independent examiners	Kindergarten self-regulation skills, including classroom engagement (β = .438, 95%CI [.031, .844], p = .035) and emotional distress (β = −.108, 95%CI [−.178, −.038], p = .003), were significantly associated with subsequent parent-reported child sports participation<br>A higher score on self-regulation skills significantly predicted lower BMI. Specifically, classroom engagement was significantly associated with fourth grade BMI (β = −.689, 95% CI [−1.132, −.067], p = .030)	*Social Cognitive Theory*<br>*optimal self-regulation may help children sustain involvement in structured sports and maintain a healthy BMI by facilitating the exercise of self-control and delay of gratification in the face of challenges and more immediate rewards.*	Being a boy, higher SES and less television viewing associated with more physical activity. Kindergarten BMI and higher SES also associated with BMI in fourth grade.
Piche et al 2019	Longitudinal	Canada	1516 from the Quebec Longitudinal Study of Child	Participation in structured and unstructured	Parents reported on their children's participation	Age 8 Depressive symptoms,	Depression symptoms assessed through the Social	Structured physical activity negatively associated with boys' depressive symptoms one year later (ß = -.09, p =0.009). Unstructured	*It is suggested by the authors that the positive influence of structured physical activity compared to*	Sex – association between structured physical activity

Author (Year)	Study Design	Country	Participants	Exposure (measure of development) and age	Exposure Measurement Instrument	Outcome and age	Outcome Measurement Instrument	Results	Mechanism - in study (normal font) or *theorized (italics)*	Factors which moderate the association
			Development	physical activity - Age 7	in structured and unstructured physical activity		Behaviour Questionnaire.	physical activity positively associated with girls' depressive symptoms (β = .07, p = .040)	*unstructured is linked to the combination of psychosocial components of structured activities (self-regulation, self-efficacy, social competencies) and biological changes brought upon by being physically active*	at age 7 and depression at age 8 found in boys but not girls.
Rudasill et al 2014	Longitudinal	USA	1156 children from the National Institute of Child Health and Human Development (NICHD) Study of Early Child Care and Youth Development (SECCYD)	Child temperament (negative emotionality at age 4½ and emotional reactivity at age 7-12)<br><br>(Student-teacher relationship - teacher perception and child perception tested as mediators)	Negative emotionality: Mothers completed eight subscales from the Children's Behaviour Questionnaire<br>Emotional reactivity: Children's emotional responses to events and environmental stimuli were rated by mothers using a measure designed for use in the	Depressive symptoms in sixth grade (age 11-12)	Mother report of their children's depressive symptoms was measured in 6th grade with the Diagnostic and Statistical Manual of Mental Disorders oriented Affective Problems subscale of the Child Behaviour Checklist	<u>Student perceptions of teacher support:</u><br>• Children with higher negative emotionality at age 4½ were rated as more emotionally reactive (β=.439, p < .001) in grades 4–6 and had more depressive symptoms (β=.182, p = .008) in grade 6. In addition, teacher support was associated with less depressive symptoms (−.465, p < .001) in grade 6.<br>• No mediating role<br><u>Teacher perception of student teacher relationship (conflict)</u><br>• Children with higher negative emotionality at age 4½ were more likely to be rated as emotionally reactive (.444, p < .001) in grades 4–6 and display more depressive symptoms	Mediators:<br>STRs were assessed using teachers' responses on the Student-Teacher Relationship Scale – teacher reported in fourth, fifth and sixth grade<br>Teacher support was measured from student responses on the School Attachment and Environment questionnaire given in sixth grade – child reported. Findings reported here indicate that one mechanism by which individuals with higher levels of emotional reactivity have more depressive symptoms	Girls more likely to rate their teachers as supportive. Teachers more likely to rate their relationships with boys as conflictual

Author (Year)	Study Design	Country	Participants	Exposure (measure of development) and age	Exposure Measurement Instrument	Outcome and age	Outcome Measurement Instrument	Results	Mechanism - in study (normal font) or *theorized (italics)*	Factors which moderate the association
					NICHD SECCYD.			(.246, p = .002) in grade 6. In addition, children showing more emotional reactivity in grades 4–6 were more likely to be rated by teachers as conflictual(.165, p < .001) . • conflict partially mediated the relationship	may be more conflict with teachers.	
Rudolph et al 2011	Longitudinal	USA	433 participating in a longitudinal study of peer victimization	Peer Victimization (static and dynamic) (Age 7-12, 2nd to 5th grade)	Children and teachers completed a revised version of the Social Experiences Questionnaire to assess children's exposure to peer victimization .	Depression symptoms and Aggressive behaviour - Age 11-12 (5th grade)	Depression symptoms - Short Mood and Feelings Questionnaire (Child report) Aggressive behaviour - Children's Social Behaviour Scale (teacher report)	Both early and increasing victimization were significantly associated with fifth-grade depressive symptoms, overt aggression, and relational aggression. Both early and increasing victimization over time more strongly predicted fifth-grade relational aggression in girls than in boys.	*Victimization prompts girls to engage in relational aggression during elementary school, and subsequently relational aggression may elicit negative responses such as peer rejection which heighten girls' risk for depressive symptoms during adolescence.*	Sex - relational agression stronger in girls
Sandstrom et al 2020	Meta-analysis	Any	8836 children	The mean age at the first BI assessment was 3.61 years	BI: defined as shyness, fear, and avoidance when faced with new stimuli	The mean age at the anxiety assessment was 10.39 years	Anxiety and specific anxiety types searched	BI significantly increased the subsequent risk of anxiety (OR = 2.80, 95% CI 2.03 to 3.86, p < 0.001 Children with BI were significantly more likely to have SAD (OR = 5.84, 95% CI 3.38 to 10.09, p < 0.001; Fig. 3), GAD (OR = 2.04, 95% CI 1.43 to 2.91, p < 0.001), and specific phobia (OR = 1.49, 95% CI 1.03 to 2.14, p = 0.03 but not separation anxiety	-	

Author (Year)	Study Design	Country	Participants	Exposure (measure of development) and age	Exposure Measurement Instrument	Outcome and age	Outcome Measurement Instrument	Results	Mechanism - in study (normal font) or *theorized (italics)*	Factors which moderate the association
Sasser et al 2017	Longitudinal	USA	356 children enrolled in a Head start programme, all children from low-income families	Intervention targetting social-emotional functioning and language-emergent literacy skills in the first year of pre-school. EF measured before and after preschool and each year to third grade (age 8)	EF assessment by trained examiners. Children assigned to either low, moderate or high EF trajectory	Third grade academic outcomes	Reading fluency, language-arts and maths (all teacher rated), children self-evaluation of reading ability	Significant effect of intervention for children with low EF trajectories, with children in the intervention group showing a more positive slope of growth in EF between preschool and third grade (d = 0.19, p = .004) and exhibiting significantly higher third-grade EF scores (d = 0.70, p = .002), compared with children in the control group. Significant intervention effects favouring children in the intervention group emerged for children with low EF trajectories on academic outcomes of: reading fluency, language arts and math performance and children's self-perceptions of their reading ability.	-	EF moderates the effect of intervention (social-emotional functioning and language-emergent literacy skills) on academic outcomes

Author (Year)	Study Design	Country	Participants	Exposure (measure of development) and age	Exposure Measurement Instrument	Outcome and age	Outcome Measurement Instrument	Results	Mechanism - in study (normal font) or *theorized (italics)*	Factors which moderate the association
Shapero et al 2013	Longitudinal	USA	958 children from the National Institute of Child Health & Human Development Study of Early Child Care and Youth Development (SECCYD)	Emotional - emotional reactivity at age 8.<br><br>(Household income and household chaos also studied.)	Emotional reactivity – mother report - 10-item questionnaire about their perceptions of how their child expresses emotions in response to events	Emotional and behavioural problems - Age 15	Adolescent Emotional and Behavioural Problems – Youth Self-Report.	Higher levels of emotional reactivity predicted higher levels of emotional and behavioural problems (B = 0.122, p <0.001). Income also predictive of the outcome. Household chaos not predictive.<br>The interaction of household chaos and childhood emotional reactivity significantly predicted increases in adolescent total problems and internalizing problems (B=0.104, P<0.01), but not externalizing problems<br>Emotional reactivity did not moderate the relationship between income and increases in problem behaviours.	*Detrimental effect of physical and psychological aspects of the home environment leading to internalizing problems.*	Household chaos is a moderator of the relationship between Temperament (emotional reactivity) and adolescent internalizing behaviour problems but income is not.
Slemming et al 2010	Longitudinal	Denmark	1336 from the Aarhus Birth cohort, Denmark	Behaviour: anxious–fearful, hyperactive–distractible, and hostile–aggressive - Age 3-4	Preschool behaviour questionnaire (PBQ) - parent report	Internalizing problems - Age 10-12	Emotional difficulties were measured at age 10–12 years with the parent-administered strength and difficulties questionnaire (SDQ)	Anxious–fearful associated with internalizing symptoms: OR 2.1, 95% CI 1.1–4.0) and hostile–aggressive associated with internalizing symptoms: OR 2.4, 95% CI 1.3–4.7).<br>Hyperactive–distractible preschool behaviour was not associated with school-age emotional difficulties when confounding factors considered.	*Hostile–aggressive behaviour may lead to internalizing disorder due to increased environmental stress, a common precursor for the two dimensions or a longitudinal transformation of one behavioural dimension into another in later childhood.*	Relationship held regardless of confounders

Author (Year)	Study Design	Country	Participants	Exposure (measure of development) and age	Exposure Measurement Instrument	Outcome and age	Outcome Measurement Instrument	Results	Mechanism - in study (normal font) or *theorized* (*italics*)	Factors which moderate the association
Straatmann et al 2018	Longitudinal	UK	10262 from the Millennium Cohort Study UK	Five central domains of a health check in England: (1) personal, social and emotional development, (2) communication and language, (3) physical health, (4) learning and cognitive development and (5) physical development and self-care) - at Age 3	Health visitor assessment at routine health check	Language ,weight, socioemotional behaviour - Age 11	Language - British Ability Scale Second Edition (BAS II) Verbal Similarities test. Weight was derived from the body mass index (BMI), using the age and sex-International Obesity Task Force cut-offs. Socio-emotional behaviour - SDQ - mother report	Model 1 - routine data only. Model 2 - routine data plus data from the five domains (exposure), model 3 - all from model 1 and 2 plus additional risk factors. Language disability, overweight/obesity and socioemotional problems identified with moderate discrimination in model 2 with (AUROC: 0.73, 95%CI 0.71 to 0.75) (AUROC: 0.73, 95%CI 0.72 to 0.74) and AUROC: 0.77, 95%CI 0.75 to 0.79, respectively. Model 2 resulted in a significant improvement over model 1, for overweight/obesity and socioemotional problems with 8.14% and 6.26% more children being correctly reclassified, respectively	-	
Sutin et al 2017	Longitudinal	Australia	4153 from the Longitudinal Study of Australian Children	Temperament - sociability, persistence, negative reactivity. Age 4-5	Parents completed a 12-item measure of temperament based on the Childhood Temperament Questionnaire.	Weight and weight attitudes and behaviour - Age 14-15	Weight – BMI and waist circumference at all ages Weight attitudes and behaviour. At ages 14–15 years, study children self-reported on several aspects of their attitudes	Persistence associated with a decreased risk of obesity OR = 0.82, CI = 0.69–0.97), overweight (OR = 0.88, CI = 0.78– 0.98) and W:H⩾0.5 (OR = 0.87, CI = 0.77–0.98). Sociability associated with an increased risk of overweight (OR = 1.10, CI = 1.01–1.20) and W:H⩾ .5 (OR = 1.10, CI = 1.01–1.21) but not with obesity risk (OR = 1.10, CI = 0.96–1.26). Negative reactivity was unrelated to risk of	*Individual differences in psychological functioning make some people more vulnerable and others more resilient and points to the importance of addressing both the individual and the environment in weight gain across the lifespan*	Girls higher on sociability had a greater fear of weight gain

Author (Year)	Study Design	Country	Participants	Exposure (measure of development) and age	Exposure Measurement Instrument	Outcome and age	Outcome Measurement Instrument	Results	Mechanism - in study (normal font) or *theorized (italics)*	Factors which moderate the association
							and behaviours.	overweight, obesity or elevated W:H. Lower persistence and higher negative reactivity were associated with greater weight concerns, restrained eating and use of unhealthy weight-management strategies at ages 14–15 years.		
Weeks et al 2014	Longitudinal	Canada	4405 children from the Canadian National Longitudinal Study of Children and Youth (NLSCY)	Verbal ability (age 4-5) and Math skills - age 7-11	Verbal Ability: Peabody Picture Vocabulary Test-Revised (PPVT-R) Math skills - children completed a Mathematics Computation Test (MCT).	Internalizing symptoms of anxiety and depression - Age 12-13 and 14-15	Questionnaire that included 7 items from the Ontario Child Health Study (OCHS-R), assessing symptoms of anxiety and depression - self-report.	Greater verbal ability at age 4–5 decreased the odds of moderate (Adj OR=0.83; CI: 0.75, 0.93) and severe (Adj OR=0.80; CI: 0.70, 0.92) internalizing symptoms at age 12-13 and decreased the odds of mild symptoms (OR=0.84; CI: 0.76, 0.92) at age 14-15. Higher maths ability at age 6–7 increased the odds of mild symptoms (Adj OR=1.21; CI: 1.05, 1.39) at age 12-13 and increased the odds of mild (Adj OR=1.44; CI: 1.21, 1.72) and severe (Adj OR=1.50; CI: 1.18, 1.92) internalizing symptoms at age 14-15.	*Hormonal changes to explain the loss of the protective effect of cognitive ability on internalizing symptoms. Children with greater cognitive ability in childhood may be better at coping with the stress of family dysfunction and chronic illness in childhood, perhaps because they are more capable of problem-solving and adapting to more stressful environments.*	Gender and family stress: Some of the effects of childhood cognitive ability varied with child gender. Also, childhood cognitive ability attenuated the effects of family dysfunction and chronic illness throughout childhood on subsequent internalizing symptoms.
Yan et al 2018	Longitudinal	USA	695 from the NICHD SECCYD study.	Emotional Wellbeing - child parent relationship - Age 6	Both fathers and mothers rated their relationships (conflict and closeness) with the	Loneliness at grades 1, 3 and 5 (age 10-11)	Loneliness and Social Dissatisfaction Questionnaire - child self-report	As parent child closeness increases, loneliness reduces The slope of father-daughter closeness was negatively associated with the slope of loneliness for	*Attachment theory.*	Gender: father-child closeness has a stronger association with girls loneliness

Author (Year)	Study Design	Country	Participants	Exposure (measure of development) and age	Exposure Measurement Instrument	Outcome and age	Outcome Measurement Instrument	Results	Mechanism - in study (normal font) or *theorized (italics)*	Factors which moderate the association
					child at Grade 1, 3, 4 and 5. Using the short form of the Child-Parent Relationship Scale			girls β = −0.46, p = .03). When father-daughter closeness declined more slowly, girls' perceived loneliness declined more quickly.		
