# Supplementary material for: Relationships between Child Development at School Entry and Adolescent Health—A Participatory Systematic Review
Source: Int J Environ Res Public Health. 2021 Nov 4;18(21):11613. doi: 10.3390/ijerph182111613 (PMC8582847; doi:10.3390/ijerph182111613)
Supplement: Supplementary file 1 [file ijerph-18-11613-s001.zip › Additional File 9 Results of Quality Assessment of Studies.pdf]

Additional File 9: Results of quality assessment of studies

**Author and Year**	**Recruitment**	**Participation***	**Description of baseline characteristics**	**Participants at follow up**	**Duration of follow up**	**Response at follow up***	**Not selective non-response***	**Exposure measurement***	**Outcome measurement***	**Analytical model***	**Adjustment for confounders***	**Measures of variability**	**Methodological Quality**
Ashford et al 2008	+	+	+	+	+	+	+	-	+	?	-	+	Moderate
Berthelsen et al 2017	+	+	+	+	+	-	+	-	+	+	+	+	High
Bornstein et al 2010	-	?	+	-	+	+	+	+	+	?	-	+	Moderate
Bornstein et al 2013	-	-	+	-	+	+	+	+	+	?	+	+	Moderate
Derks et al 2019	+	?	+	+	+	+	+	+	+	+	+	+	High
Duchesne et al 2010	+	+	+	+	-	-	+	+	+	+	+	+	High
Fine et al 2003	?	+	+	+	+	+	+	+	+	+	-	-	High
Glaser et al 2011	+	+	+	+	+	+	+	+	+	+	+	+	High
Gregory et al 2020	+	+	+	+	+	-	+	+	+	+	-	+	Moderate
Hay et al 2003	+	-	+	+	+	+	-	+	+	+	-	+	Low
Hooper et al 2003	+	?	+	-	+	?	?	-	+	+	-	+	Low
Howard et al 2018	+	+	+	+	+	-	+	+	+	+	+	+	High
Howes et al 2000	+	+	+	-	-	-	-	+	+	+	-	+	Low
Jaspers et al 2010	+	+	+	+	+	+	+	-	+	+	-	+	Moderate
Lecompte et al 2014	-	-	-	+	+	+	-	+	+	?	-	+	Low
Lee et al 2017	+	+	+	+	+	?	-	-	+	+	+	+	Moderate
Louise at al 2012	+	-	+	+	+	+	-	+	+	+	-	+	Moderate
McKenzie et al 2002	-	+	+	+	+	-	?	-	+	+	-	+	Low

Author and Year	Recruitment	Participation*	Description of baseline characteristics	Participants at follow up	Duration of follow up	Response at follow up*	Not selective non-response*	Exposure measurement*	Outcome measurement*	Analytical model*	Adjustment for confounders*	Measures of variability	Methodological Quality
Meagher et al 2009	-	?	+	+	+	+	-	-	+	?	-	+	Low
Nelson et al 2018	+	?	+	+	+	+	-	+	+	+	+	+	Moderate
Pagani et al 2014	+	-	+	+	+	-	+	+	?	+	-	+	Moderate
Pedersen et al 2007	+	+	+	+	+	-	+	-	+	+	-	-	Low
Piche et al 2012	+	-	-	+	+	+	+	+	-	+	-	+	Moderate
Piche et al 2019	+	-	-	+	+	?	+	-	+	+	+	+	Low
Rudasill et al 2014	+	+	+	+	+	-	+	-	+	+	-	+	Moderate
Rudolph et al 2011	-	+	+	-	+	+	-	-	+	+	-	-	Low
Sandstrom et al 2020	*Study design is Meta-Analysis: AMSTAR used as quality assessment tool*												Low
Sasser et al 2017	+	+	+	+	+	+	+	-	+	+	-	-	Moderate
Shapero et al 2013	+	-	+	-	+	+	-	+	+	+	-	+	Moderate
Slemming et al 2010	+	-	+	+	+	+	-	-	-	+	+	+	Moderate
Straatmann et al 2018	+	-	-	-	+	+	+	+	+	+	+	+	High
Sutin et al 2017	+	+	+	+	+	+	+	-	+	+	-	+	High
Weeks et al 2014	+	-	+	+	+	+	?	-	+	+	+	+	Moderate
Yan et al 2018	+	+	+	-	+	?	+	-	+	+	+	+	Moderate
