# Supplementary material for: Relationships between Child Development at School Entry and Adolescent Health—A Participatory Systematic Review
Source: Int J Environ Res Public Health. 2021 Nov 4;18(21):11613. doi: 10.3390/ijerph182111613 (PMC8582847; doi:10.3390/ijerph182111613)

Additional File 2: Conceptual Diagram

How does development in the early years of school age children affect health in adolescence in the context of socioeconomic inequality?

Early-Childhood to Early Adolescence (age 3-15)

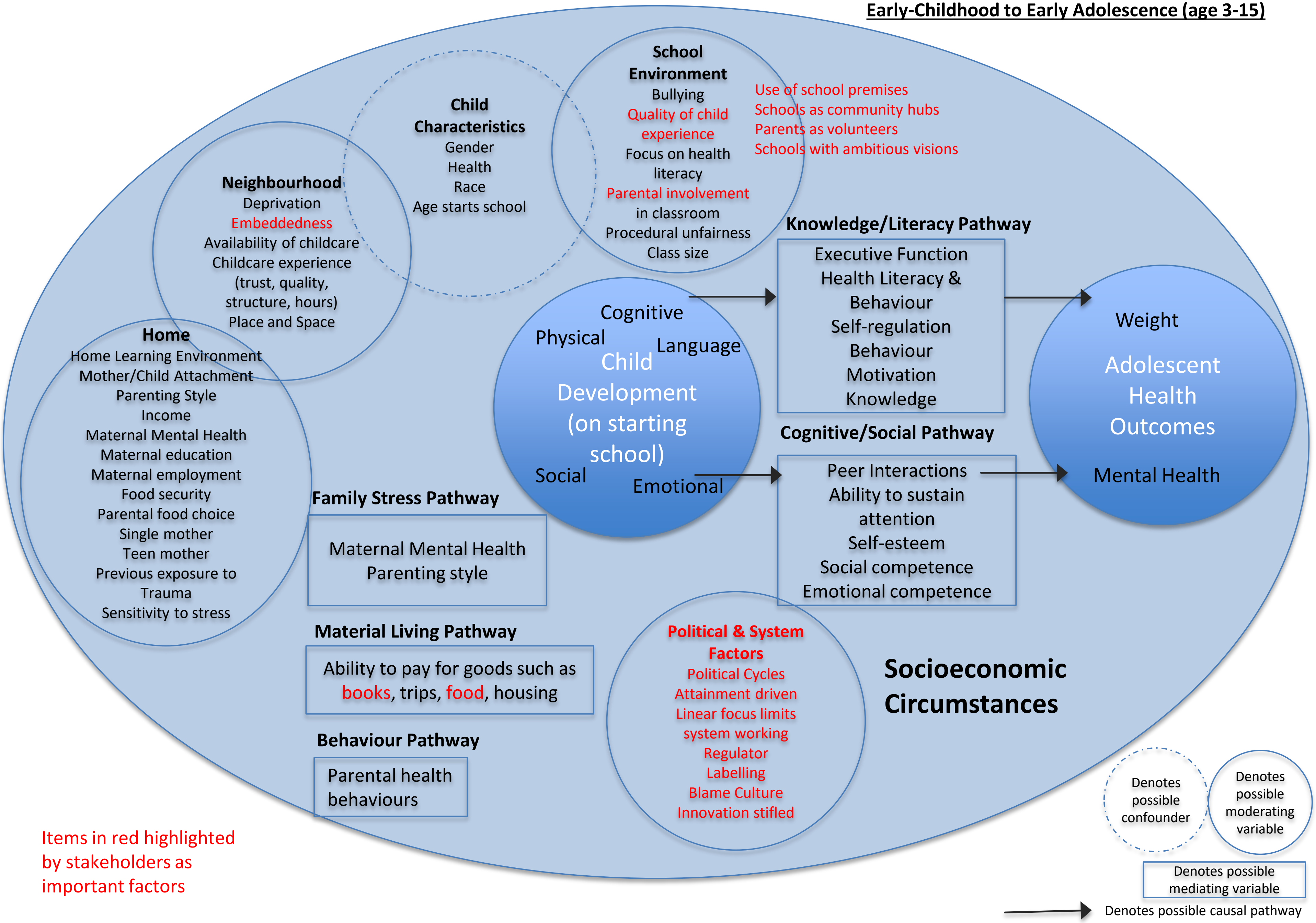

Supplement: Supplementary file 1 [file ijerph-18-11613-s001.zip › Additional flie 2 Initial Conceptual Model.pdf]
